# Management and 30-Day Mortality of Acute Coronary Syndrome in a Resource-Limited Setting: Insight From Ethiopia. A Prospective Cohort Study

**DOI:** 10.3389/fcvm.2021.707700

**Published:** 2021-09-17

**Authors:** Korinan Fanta, Fekede Bekele Daba, Elsah Tegene Asefa, Tsegaye Melaku, Legese Chelkeba, Ginenus Fekadu, Esayas Kebede Gudina

**Affiliations:** ^1^Department of Clinical Pharmacy, Institute of Health, Jimma University, Jimma, Ethiopia; ^2^Department of Internal Medicine, Institute of Health, Jimma University, Jimma, Ethiopia; ^3^Department of Pharmacology and Clinical Pharmacy, College of Health Science, Addis Ababa University, Addis Ababa, Ethiopia; ^4^School of Pharmacy, Faculty of Medicine, The Chinese University of Hong Kong, Shatin, Hong Kong, SAR China; ^5^Department of Clinical Pharmacy, Institute of Health Sciences, Wollega University, Nekemte, Ethiopia

**Keywords:** acute coronary syndrome, management, mortality, sub-Saharan Africa, myocardial infarction

## Abstract

**Background:** Despite the fact that the burden, risk factors, and clinical characteristics of acute coronary syndrome (ACS) have been studied widely in developed countries, limited data are available from sub-Saharan Africa. Therefore, this study aimed at evaluating the clinical characteristics, treatment, and 30-day mortality of patients with ACS admitted to tertiary hospitals in Ethiopia.

**Methods:** A total of 181 ACS patients admitted to tertiary care hospitals in Ethiopia were enrolled from March 15 to November 15, 2018. The clinical characteristics, management, and 30-day mortality were evaluated by ACS subtype. The Cox proportional hazards model was used to determine the predictors of 30-day all-cause mortality. A *p*-value < 0.05 was considered statistically significant.

**Results:** The majority (61%) of ACS patients were admitted with ST-segment elevation myocardial infarction (STEMI). The mean age was 56 years, with male predominance (62.4%). More than two-thirds (67.4%) of patients presented to hospital after 12 h of symptom onset. Dyslipidemia (48%) and hypertension (44%) were the most common risk factors identified. In-hospital dual antiplatelet and statin use was high (>90%), followed by beta-blockers (81%) and angiotensin-converting enzyme inhibitors (ACEIs; 72%). Late reperfusion with percutaneous coronary intervention (PCI) was done for only 13 (7.2%), and none of the patients received early reperfusion therapy. The 30-day all-cause mortality rate was 25.4%. On multivariate Cox proportional hazards model analysis, older age [hazard ratio (HR) = 1.03, 95% CI = 1.003–1.057], systolic blood pressure (HR = 0.99, 95% CI = 0.975–1.000), serum creatinine (HR = 1.32, 95% CI = 1.056–1.643), Killip class > II (HR = 4.62, 95% CI = 2.502–8.523), ejection fraction <40% (HR = 2.75, 95% CI = 1.463–5.162), and STEMI (HR = 2.72, 95% CI = 1.006–4.261) were independent predictors of 30-day mortality.

**Conclusions:** The 30-day all-cause mortality rate was unacceptably high, which implies an urgent need to establish a nationwide program to reduce pre-hospital delay, promoting the use of guideline-directed medications, and increasing access to reperfusion therapy.

## Introduction

Globally, cardiovascular disease (CVD) remains the leading cause of morbidity and mortality (1). In 2017 alone, CVD was responsible for around 17.8 million deaths worldwide, of which more than 75% were in low- and middle-income countries (2). Among CVDs, ischemic heart disease (IHD) is the most prevalent and caused ~9 million deaths globally in 2017 (2). Advancement in the emergency treatment of acute coronary syndrome (ACS) and primary and secondary prevention measures have resulted in a significant decrease in age-adjusted mortality from IHD in developed countries (3, 4). Nevertheless, low- and middle-income countries are markedly suffering from IHD mortality (2).

Although IHD was previously considered rare in sub-Saharan Africa (SSA), currently, it is one of the top five causes of mortality and morbidity in the region (4–6). This demonstrates that rapid epidemiological transition and an increase in CVD burden are taking place in the region (5). Furthermore, the major risk factors of IHD, such as hypertension, abdominal obesity, diabetes, dyslipidemia, and smoking, are increasing as a result of lifestyle changes, urbanization, and increased life expectancy (7).

Unfortunately, the majority of SSA countries are still in a pre-reperfusion era and have no access to affordable effective medications (8). Additionally, large prospective registry data on ACS management and mortality in the region are limited to the ACESS-South African Study, which included only private healthcare centers with interventional cardiology (9). As a result, this finding might not represent most of the resource-limited setups in SSA countries, including Ethiopia.

According to a few retrospective analyses of patients' medical records conducted in Ethiopia (10–12), so far, the in-hospital mortality rate from ACS was 14.4–29.4%. Although the figures are alarming, all the studies were based on a small sample size and a retrospective review of patient data. Therefore, we prospectively assessed the clinical characteristics, management, and 30-day mortality of ACS patients admitted to tertiary care hospitals in Ethiopia to increase awareness on the current management and clinical outcomes of ACS patients.

## Materials and Methods

### Study Design and Settings

A prospective cohort study was carried out at Jimma University Medical Center (JUMC) and Saint Peter Specialized Hospital (SPSH) in Ethiopia from March 15 to November 15, 2018. JUMC serves as a referral center for catchment areas in the southwest part of Ethiopia with a population of around 15 million. The hospital has a range of specialty wards, such as pediatric, internal medicine (cardiac unit, stroke unit, pulmonology unit, and renal and general medical ward), surgery, and other specialty services. JUMC is equipped with an electrocardiogram, transthoracic echocardiogram, computed tomography, and other diagnostic equipment. The hospital has two cardiologists and 15 internists during the study period. SPSH is located in the capital city (Addis Ababa), and it has a cardiac center equipped with a cardiac catheterization lab. The hospital has three cardiologists and 10 internists during the study period.

### Study Population

All consecutive adult patients (>18 years of age) who presented with new or worsening symptoms suggestive of myocardial ischemia were enrolled. Cardiac biomarkers (troponin I and/or creatine kinase myocardial band) and electrocardiogram were performed for all patients. ACS was diagnosed by treating cardiologists based on the third universal definition of myocardial infarction (13). Patients who died before evaluation and confirmation of ACS diagnosis, readmitted patients (enrolled previously), those who declined informed consent, and those unable to attend the 30-day follow-up were excluded from this study, as previously described (14). Therefore, the patient profiles in the present findings share similarities with previously published articles of the same project. The patients were classified as having ST-segment elevation myocardial infarction (STEMI) or non-ST-segment elevation ACS (NSTE-ACS) (i.e., non-ST elevation myocardial infarction or unstable angina) (15, 16). The patients were followed at the Cardiac Care Unit and the General Internal Medicine ward of each hospital. All the study protocols were approved by the Institutional Review Board (IRB) of Jimma University, Institute of Health (IHRPGD/193/18), before starting data collection.

### Data Collection

The data were collected by four trained health professionals (nurses and medical interns) using a pre-tested structured questionnaire. Data collectors extracted relevant data from active medical records of patients and interviewed patients when necessary. The data collection tool was developed based on previous ACS registries (17, 18). The data collection tool contains five sections (demographic profile, risk factors, clinical presentations, treatments, and outcomes). All relevant patient cases were recorded carefully by following patients from admission to discharge or death. Important medical and behavioral history was collected by interviewing the patients, when necessary. Operational definitions of the risk factors or comorbid conditions assessed in this study are available in the Supplementary Material. Initiation of guideline-directed medications was assessed during the hospital stay and at discharge. The primary end point of the study was 30-day all-cause mortality. Data on the mortality were collected by following patients on a daily basis from hospital admission to 30 days or death prospectively. The physician's death summary notes were reviewed for patients who died in the hospital. For those who died after discharge, family members/caregivers were contacted through telephone to confirm the death.

### Statistical Analysis

Complete case records of patients were entered into EpiData, version 4.2, and the data were analyzed using the Statistical Package for Social Science (SPSS), version 23 (IBM, Armonk, NY, USA). Categorical data were presented as frequency and percentage, while normally distributed continuous variables were reported as the mean ± standard deviation (SD) and non-normally distributed data as medians with interquartile ranges (IQRs). Categorical variables were compared using the chi-squared test or Fisher's exact test, as necessary. Student's *t*-test or the Mann–Whitney *U*-test was performed to compare continuous variables, as required. The Kaplan–Meier curve was used to summarize time-to-event distributions according to the ACS subtypes and compared by a log-rank test. Multivariable Cox proportional hazards analyses were done to identify independent predictors of 30-day all-cause mortality. Significant variables from univariate analysis (*p* < 0.05) and other relevant variables were included in the multivariable analysis. We checked the proportional hazards assumption by plotting residuals against time. The backward stepwise method was chosen and variables with a two-sided *p*-value < 0.05 were considered statistically significant. The sample size was calculated using Epitools epidemiological calculators (available at http://epitools.ausvet.com.au.) based on data from a prior study on ACS in Ethiopia (12). The minimum sample size calculated was 156 patients (78 patients per group). However, we included all eligible patients admitted to the hospitals during the study period (181 patients).

## Results

**Figure 1:**
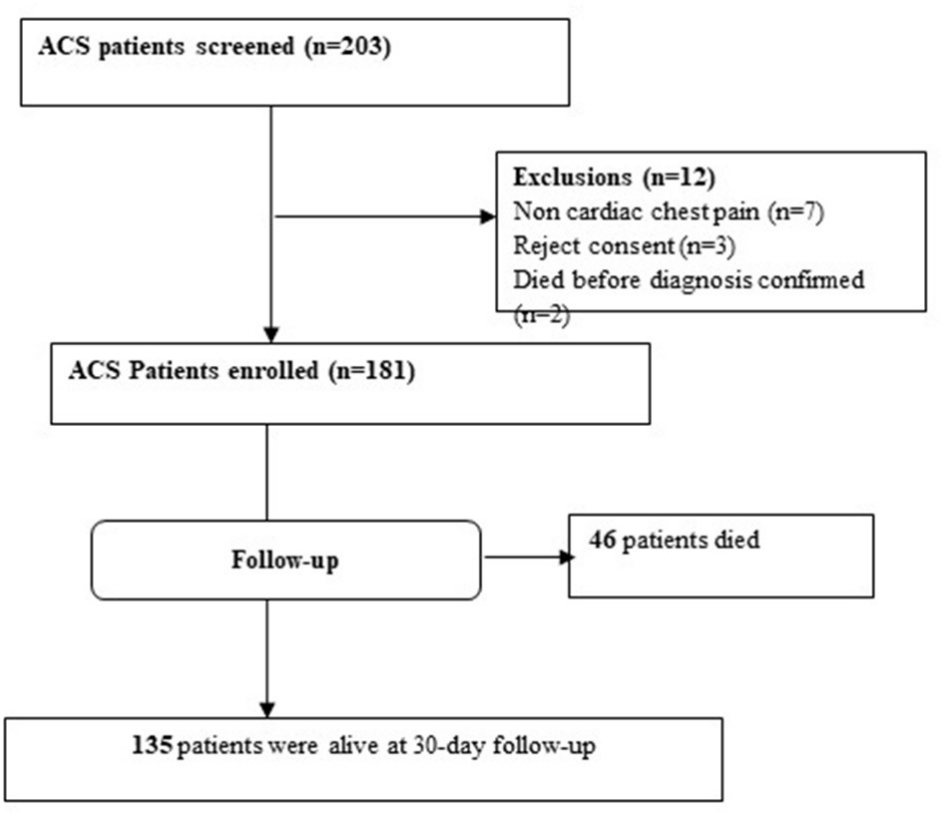
Study flow chart of ACS patients admitted to tertiary hospitals in Ethiopia during the study period.

Among the 203 patients presenting with ACS, 181 patients fulfilled the inclusion criteria. The enrolled patients were followed for 30 days starting from the first day of admission. Among the 181 patients followed, 46 (25.4%) died during follow-up and 135 (74.6%) were alive at the end of the follow-up period (Figure 1).

### Demographics and Medical History

Among the 181 ACS patients enrolled, 113 (62.4) were males and the mean age was 55.8 ± 11.9 years. The majority of the patients presented with STEMI (61%) and NSTE-ACS (39%). About two-thirds (63%) of the patients were referred by primary healthcare, and only 17 (9.4%) patients received ambulance service. One in four patients (26.5%) had insurance coverage for services or medications available at the public hospitals.

Hypertension was identified in around 44%, and almost half (48%) of the patients had dyslipidemia. One in four participants had a prior history of diabetes and myocardial infarction. The less common risk factors were smoking (16%), prior history of chronic kidney disease (9%), and HIV/AIDS (7%) (Table 1).

**Table 1:** Baseline clinical characteristics of the study participants by ACS subtype.

**Variables**	**STEMI (*n* = 111)**	**NSTE-ACS (*n* = 70)**	**Total (*N* = 181)**	***p*-value**
Sex (male), *n* (%)	70 (63)	43 (61.3)	113 (62.4)	0.825
Age (years), ±SD	55.2 ±11.4	56.9 ±12.7	55.8 ± 11.9	0.349
**Residence**, ***n*** **(%)**				
Urban	85 (76.6)	52 (74.3)	137 (75.7)	0.726
Rural	26 (23.4)	18 (25.7)	44 (24.3)	
**Educational status**, ***n*** **(%)**				
No formal education	60 (54.0)	39 (55.7)	99 (54.7)	0.827
Formal education	51 (45.9)	31 (44.3)	82 (45.3)	
**Occupation**, ***n*** **(%)**				
Employed	24 (21.6)	19 (27.1)	43 (23.7)	0.442
Farmers/labor workers	55 (49.5)	28 (40)	83 (45.9)	
Unemployed	32 (28.8)	23 (32.8)	55 (30.4)	
**Admission status**, ***n*** **(%)**				
Referral	71 (64.0)	43 (61.4)	114 (63.0)	0.713
Direct admission	40 (36.0)	27 (38.6)	67 (37.0)	
**Mode of transportation**, ***n*** **(%)**				
Ambulance	13 (11.7)	4 (5.7)	17 (9.4)	0.178
Taxi/other automobile	98 (88.3)	66 (94.3)	164 (90.6)	
**Payment methods**, ***n*** **(%)**				
Insurance^a^	35 (31.5)	13 (18.6)	48 (26.5)	0.054
Out of pocket	76 (68.5)	57 (81.4)	133 (73.5)	
**Risk factors**, ***n*** **(%)**				
Hypertension	47 (42.3)	32 (45.7)	79 (43.6)	0.656
Diabetes mellitus	27 (24.3)	19 (27.1)	46 (25.6)	0.671
Dyslipidemia	54 (48.6)	33 (47.1)	87 (48.1)	0.843
Family history of CVD	23 (20.7)	22 (31.4)	45 (24.9)	0.105
Prior MI	22 (19.8)	15 (21.4)	37 (20.4)	0.794
CKD	10 (9.0)	7 (10.0)	17 (9.4)	0.824
HIV/AIDS	4 (3.6)	8 (11.4)	12 (6.6)	0.039^*^
Smoking	16 (14.4)	13 (18.6)	29 (16.0)	0.458
Chewing khat^b^	23 (20.7)	19 (27.1)	42 (23.2)	0.319

*CVD, cardiovascular disease; CKD, chronic kidney disease; NSTE-ACS, non-ST-segment elevation acute coronary syndrome; MI, myocardial infarction; SD, standard deviation; STEMI, ST-segment elevation myocardial infarction*.

* *p < 0.05 (statistically significant)*.

a *Only cover medications and services available at a public hospital*.

b *Catha edulis (khat) is a natural stimulant green leafy plant commonly grown in the Horn of Africa*.

### Clinical Presentations and Diagnostic Evaluation

The median time from symptom onset to hospital presentation was 26 h (range, 10–43 h). More than one-third of the patients (36%) had atypical presentations of atypical chest pain and dyspnea. Patients with ST-segment myocardial infarction were more likely to present with Killip class > II (27 vs. 8.6%, *p* = 0.002) and positive cardiac biomarkers (80 vs. 60%, *p* = 0.003) than did NSTE-ACS patients. Intermediate and high Global Registry of Acute Coronary Events (GRACE) risk scores were recorded in 35 and 20%, respectively, without significant difference between ACS subtypes. Transthoracic echocardiography was done for almost all patients during their hospital stay, and 38.7% of the patients had ejection fraction <40%. Diagnostic coronary angiogram was done in 79 (44%) patients (Table 2).

**Table 2:** Clinical presentations and diagnostic evaluations by ACS subtype.

**Variables**	**STEMI (*n* = 111)**	**NSTE-ACS (*n* = 70)**	**Total (*N* = 181)**	***p*-value**
**Presentations**				
Symptom onset to admission (h)^a^	26 (8–48)	26.5 (12–40)	26 (10–43)	0.925
<12 h, *n* (%)	40 (36.0)	19 (27.1)	59 (32.6)	0.241
≥12 h, *n* (%)	71 (64.0)	51 (72.9)	122 (67.4)	
**Symptom at arrival**, ***n*** **(%)**				
Typical angina pain	73 (65.3)	40 (57.1)	113 (62.4)	0.243
Atypical presentation	31 (27.9)	34 (48.6)	65 (35.9)	
Heart rate (bpm)^b^	91.6 ± 21.7	92.5 ± 27.7	92.0 ± 24.1	0.966
Systolic BP (mmHg)^b^	130.3 ± 30.6	129.7 ± 26.2	129.7 ± 29.2	0.888
Creatinine > 2 mg/dl, *n* (%)	16 (14.4)	9 (12.8)	25 (13.8)	0.767
Killip class > II, *n* (%)	33 (29.7)	10 (14.3)	36 (23.8)	0.017^*^
**GRACE risk score**, ***n*** **(%)**				
Low	45 (50.0)	35 (40.5)	80 (44.2)	0.458
Intermediate	42 (31.4)	22 (37.8)	64 (35.4)	
High	24 (18.6)	13 (21.6)	37 (20.4)	
**Key investigation**, ***n*** **(%)**				
Positive cardiac biomarkers	89 (80.2)	42 (60)	131 (72.4)	<0.001^*^
Diagnostic angiogram	52 (46.8)	27(38.6)	79 (43.6)	0.274
Ejection fraction <40%	42 (37.8)	28 (40.0)	70 (38.7)	0.771

*IQR, interquartile range; NSTE-ACS, non-ST-segment elevation acute coronary syndrome; STEMI, ST-segment elevation myocardial infarction; GRACE, Global Registry of Acute Coronary Events*.

* *p < 0.05 (statistically significant)*.

a *Expressed as median and interquartile range*.

b *Expressed as mean and standard deviations*.

### In-Hospital Management and Outcomes

About 73% of ACS patients received dual antiplatelet therapy (aspirin + clopidogrel) within the first 24 h of hospital admission; about 75% received statins. Beta-blockers and angiotensin-converting enzyme inhibitors (ACEIs)/angiotensin receptor blockers (ARBs) were given to 73 and 66%, respectively. Regarding in-hospital treatment, almost all (>98%) patients received aspirin and statins. Dual antiplatelet therapy (aspirin + clopidogrel) was given to >90% across all the ACS subtypes. Beta-blockers, heparin, and ACEIs/ARBs were administered to 81, 76, and 72%, respectively. More STEMI patients (49%) received morphine compared to NSTE-ACS patients (31%, *p* = 0.022) (Table 3). At discharge, aspirin (97%) and statins (94%) were the two most commonly prescribed medications, followed by beta-blockers (80%), clopidogrel (75.7%), and ACEIs/ARBs (75.7%) (Table 3). The all-cause in-hospital mortality was 20.4%, with a significant difference between STEMI (26.1%) and NSTE-ACS (11.4%, *p* = 0.017) patients. The mean hospital stay was 12.4 ± 6.3 days.

**Table 3:** In-hospital management and outcomes among patients admitted with ACS.

**Management**	**STEMI**	**NSTE-ACS**	**Total**	***p*-value**
No. of patients	*n* = 111	*n* = 70	*N* = 181	
**Within the first 24 h**, ***n*** **(%)**				
Aspirin	92 (82.9)	48 (68.6)	140 (77.3)	0.025^*^
DAPT^a^	88 (79.3)	44(62.9)	132 (72.9)	0.015^*^
Statins	90 (81.1)	45 (64.3)	135 (74.6)	0.011^*^
Beta-blockers	81 (73.0)	41(58.6)	122 (67.4)	0.044^*^
ACEIs/ARBs	73 (65.8)	38 (54.3)	111 (61.3)	0.112
**During hospital stay**, ***n*** **(%)**				
Aspirin	111 (100.0)	69 (98.6)	180 (99)	0.207
DAPT^a^	103 (92.8)	63 (90.0)	166 (92)	0.507
Statins	109 (98.2)	69 (98.6)	178 (98)	0.848
Beta-blockers	90 (81.1)	56 (80.0)	146 (81)	0.858
ACEIs/ARBs	84 (75.7)	47 (67.1)	131 (72)	0.211
Any heparin	86 (77.5)	52 (74.3)	138 (76)	0.623
Nitrates	32 (28.8)	21 (30.0)	53 (29)	0.818
Morphine	54 (48.6)	22 (31.4)	76 (42.0)	0.022^*^
Diuretics	59 (53.2)	38 (34.2)	97 (53.6)	0.882
**Reperfusion therapy**				
Fibrinolytic medications	0	0	0	–
PCI^b^	10 (9)	3 (3.4)	13 (7.2)	0.231
No. of patients	*n* = 82	*n* = 62	*N* = 144	
**Discharge medications**, ***n*** **(%)**				
Aspirin	82 (100.0)	58 (93.5)	140 (97.2)	0.015^*^
Clopidogrel	69 (84.1)	40 (64.5)	109 (75.7)	0.014^*^
Beta-blockers	72 (87.8)	48 (77.4)	120 (83.3)	0.511
ACEIs/ARBs	67 (81.7)	42 (67.7)	109 (75.7)	0.379
Statin	80 (97.6)	58 (93.5)	138 (95.8)	0.915
Spironolactone	17 (20.7)	12 (19.4)	29 (20.1)	0.817
**In-hospital outcomes**, ***n*** **(%)**				
All-cause mortality	29 (26.1)	8 (11.4)	37 (20.4)	0.017
Cardiovascular death^c^	22 (19.8)	5 (7.1)	27 (14.9)	0.020
Hospital stay (days)^d^	12.4 ± 7.0	11.1 ± 5.1	12.4 ± 6.3	0.903
30-day mortality	35 (31.5)	11 (15.7)	46 (25.4)	0.017^*^

*ACEIs, angiotensin-converting enzyme inhibitors; ARBs, angiotensin receptor blockers; DAPT, dual antiplatelet therapy; NSTE-ACS, non-ST-segment elevation acute coronary syndrome; PCI, percutaneous coronary intervention; STEMI, ST-segment elevation myocardial infarction*.

* *p < 0.05 (statistically significant)*.

a *Aspirin + clopidogrel*.

b *All performed after 12 h of symptom onset (late reperfusion)*.

c *Composite of fatal cardiovascular shock, cardiac arrest, arrhythmia, acute heart failure, and myocardial infarction*.

d *Expressed as the mean and standard deviation*.

### Thirty-Day Mortality by ACS Subtype

**Figure 2:**
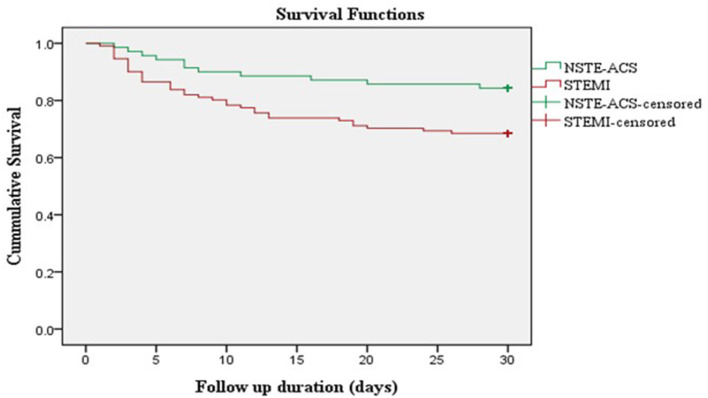
Kaplan Meier survival curve of ACS patients stratified according to ACS subtypes. NSTE-ACS, non-ST-elevation acute coronary syndrome; STEMI, ST-elevation myocardial infarction.

A cohort of 181 patients was followed up for a total of 4,419 person-days. During the 30-day follow-up period, 46 (25.4%) patients died. The overall incidence rate of mortality in ACS patients was 10.41 per 1,000 person-days. The incidence rate of mortality in STEMI patients was 13.64 per 1,000 person-days and in NSTE-ACS patients was 5.94 per 1,000 person-days. Kaplan–Meier survival analysis showed a higher risk of 30-day mortality among patients presenting with STEMI compared to non-STEMI patients (Figure 2).

### Predictors of 30-Day Mortality

On multivariate Cox proportional hazards model analysis, the risk of 30-day mortality increased as the patient's age increased [hazard ration (HR) = 1.03, 95% CI = 1.003–1.057]. Similarly, as admission serum creatinine increased by 1 mg/dl, the hazard of death increased by 32% (HR = 1.32, 95% CI = 1.056–1.643). Patients presenting with Killip class > II had an almost five times increased risk of 30-day mortality compared to those presenting with Killip class ≤ II (HR = 4.62, 95% CI = 2.502–8.523). Likewise, patients with left ventricular ejection fraction (LVEF) <40% had a three times increased hazard of 30-day mortality compared to those presenting with preserved ejection fraction (HR = 2.75, 95% CI = 1.463–5.162). Lastly, patients presenting with STEMI had an approximately three times increased risk of mortality compared to those who presented with NSTE-ACS (HR = 2.72, 95% CI = 1.006–4.261). The only variable associated with a lower risk of 30-day mortality was admission systolic blood pressure (HR = 0.99, 95% CI = 0.975–1.000) (Table 4).

**Table 4:** Predictors of 30-day all-cause mortality among patients admitted with ACS.

**Variables**	**30-day all-cause mortality**			
	**Univariate analysis**		**Multivariate analysis**	
	**Unadjusted HR (95% CI)**	***p*-value**	**Adjusted HR (95% CI)**	***p*-value**
Age (per year)	1.04 (1.09–1.06)	0.007	1.03 (1.003–1.057)	0.028^*^
Heart rate (bpm)	1.01 (1.004–1.023)	0.005	1.00 (0.990–1.013)	0.810
Systolic BP (per mmHg)	0.99 (0.98–1.00)	0.047	0.99 (0.975–1.000)	0.048^*^
Creatinine (per mg/dl)	1.32 (1.10–1.6)	0.003	1.32 (1.056–1.643)	0.015^*^
Positive cardiac enzyme	3.10 (0.75–12.79)	0.118	0.93 (0.144–6.041)	0.941
ST-segment deviation	4.19 (1.30–13.51)	0.016	1.30 (0.343–4.937)	0.700
Killip class > II	6.6 (3.64–11.87)	<0.001	4.62 (2.502–8.523)	<0.001^*^
STEMI vs. NSTE-ACS	2.22 (1.13–4.39)	0.021	2.72 (1.006–4.261)	0.048^*^
Ejection fraction <40%	2.89 (1.58–5.16)	0.001	2.75 (1.463–5.162)	0.002^*^

*BP, blood pressure; HR, hazard ratio; NSTE-ACS, non-ST-segment elevation acute coronary syndrome; STEMI, ST-segment elevation myocardial infarction*.

* *p < 0.05*.

## Discussion

Contemporary epidemiological data on ACS from SSA countries are based on a retrospective review of patients charts, but few studies have reported post-discharge outcomes of ACS patients (9, 19). In the present study, we prospectively recorded and analyzed the clinical features, management patterns, and short-term outcomes of ACS patients admitted to tertiary care hospitals in Ethiopia. The majority of our study participants presented with STEMI in their mid-50s, with a male predominance. More than two-thirds (67.4%) of the patients were admitted to the hospitals after 12 h of symptom onset. The overall use of guideline-directed medications was suboptimal, particularly during the first 24 h of hospital admission. The reperfusion rate with fibrinolytic therapy or PCI was negligible due to the lack of medications and PCI-capable centers. The all-cause 30-day mortality rate in the present study was significantly high.

We observed a high case of STEMI (61%), contrary to studies from developed countries that reported less than half having STEMI (17, 20, 21). However, our finding was similar to studies from SSA countries and large registries from India, which reported STEMI as the most common ACS diagnosis (22–24). The younger age at presentation (mean age, 56 years) in our study is consistent with prior findings from Africa, but a decade younger compared with those in developed countries (17, 20). This younger age at first ACS presentation in our study and in other SSA countries is most likely due to earlier acquisition of IHD risk factors (7, 25).

The present study also demonstrated high rates of dyslipidemia (48%), hypertension (44%), and diabetes (26%), while the smoking rate (16%) was relatively low. Compared with the two previous studies conducted among ACS patients in Ethiopia (11, 12), the rate of dyslipidemia was increased in the present study, whereas the other risk factors followed a similar pattern. History of hypertension was the most common risk factor identified in the two prior studies (11, 12) and in the current study. The INTERHEART Study (7), which included nine SSA countries in a multi-continental case–control study, also demonstrated that hypertension was significantly associated with myocardial infarction among a subgroup of black Africans compared to the general study population. Strategies focused on primary prevention of the risk factors of IHD, particularly hypertension, are important to decrease the increasing burden of CVD in SSA.

In this study, about 23% of ACS patients chew khat (*Catha edulis*), which is known to be associated with myocardial infraction and other cardiovascular diseases (26, 27). The prevalence of khat chewing observed in the present study was similar to that of the general population in Ethiopia (12% for females and 27% for males) (28). Although the present study did not find a significant association between khat chewing and adverse cardiovascular outcomes, previous studies have reported that khat chewing is independently associated with increased risk of stroke and mortality among ACS patients (29). Given the widespread practice of khat chewing in East Africa and Arabian countries, creating awareness regarding its cardiovascular risk is essential.

The use of guideline-directed in-hospital and discharge medications such as aspirin, second antiplatelet (clopidogrel), statins, beta-blockers, and ACEIs was almost comparable to that of other studies (17, 18, 21, 24, 30). However, as we have described in our previous study (14), a timely initiation of these guideline-directed medications still needs improvement. During the first 24 h of hospital admission, patients presenting with STEMI more likely received guideline-directed medications compared to those who presented with NSTE-ACS. This might be due to the fact that the majority of patients with STEMI presenting with typical angina pain lack local/national guidelines for the emergency management of ACS.

Only 13 (7.2%) of the patients received late reperfusion therapy (all performed after 12 h of symptom onset). None of the patients received early reperfusion therapy with PCI or fibrinolytic medications. The reason for the lack of early reperfusion was the lack of fibrinolytic therapy in public hospitals (even the relatively cheap streptokinase was not available at the time of this study). The cardiac catheterization laboratory was newly founded at the time of the study, with limited interventional cardiologists, and excessive pre-hospital delay and the in-hospital triage delay significantly contributed to suboptimal reperfusion. On top of these barriers, the affordability of cardiac catheterization and reperfusion therapy is also challenging in low-income countries like Ethiopia. Currently, there are only two PCI-capable public hospitals in Ethiopia (31).

The all-cause in-hospital mortality rate in the current study (20.4) was relatively comparable to that in the ACS registry from Kenya (22) and slightly lower than those in previous studies conducted in Ethiopia by Desta et al. (11) and Bogale et al. (12), which reported in-hospital mortality rates of 17, 24.5, and 27.4%, respectively. Nevertheless, the 30-day mortality rate observed in the present study was significantly higher compared to those of prior prospective ACS registries from South Africa (9), India (23), and six Middle East countries (30), which reported 30-day mortality rates of 3.6, 6.7, and 7.2%, respectively. This significant difference in mortality could be due to differences in the acute management of ACS, particularly due to the very late presentation and lack of early reperfusion therapy with PCI or fibrinolytic therapy in the present study.

The predictors of 30-day mortality identified in the present study were older age, admission systolic blood pressure, serum creatinine, Killip class >II, low ejection fraction (<40%), and STEMI. The prognostic value of all the above predictors was determined in the GRACE study (32), except for low ejection fraction and presentation with STEMI. Several studies have also validated the importance of the GRACE score in predicting short-term and long-term mortality from ACS (33–35). Our findings highlight that the GRACE score could be useful to stratify ACS patients even in non-PCI-cable centers. Age, serum creatinine, and ejection fraction (ACE) were also identified as predictors of short-term all-cause mortality after PCI (36–38). These findings imply that the prognostic value of the above variables can be utilized even in patients treated with medications only.

In the present study, patients diagnosed with STEMI had around a three-fold increase in 30-day mortality compared to those who presented with NSTE-ACS. The poor outcome of patients diagnosed with STEMI in the present study was mostly due to excessive pre-hospital delay and lack of coronary revascularization during index ACS admission. Immediate reperfusion with either PCI or fibrinolytic therapy is essential among ACS patients, particularly in the case of STEMI (39, 40).

The findings of this study should be interpreted in light of its strength and weakness. This study is one of the few prospective cohort studies on ACS patients in SSA. We have collected accurate and reliable data on patient demographics, management approaches, and outcomes by following patients on a daily basis at two tertiary hospitals in Ethiopia. Hence, the findings of this study demonstrate key areas that need improvement in order to reduce the mortality in ACS patients. As a weakness, the sample size used was small compared to western ACS registries due to the short enrollment period and resource constraints. Another limitation was the observational nature of the study design, which limits our ability to evaluate causation and the possibility of missing important confounders. Lastly, our study was mostly limited to patients treated only with medication due to the lack of fibrinolytic therapy and interventional cardiology services.

## Conclusion

In conclusion, our study shows that in SSA countries, particularly in Ethiopia, ACS patients present at a younger age with a high rate of STEMI than do patients in developed countries. The use of guideline-directed in-hospital and discharge medications was comparable to that of registries from developed countries, although the reperfusion therapy rate was almost zero. The 30-day all-cause mortality rate was unacceptably high, which implies an urgent need to establish a nationwide program that reduces pre-hospital delay and increases access to reperfusion therapy.

## Data Availability Statement

The original contributions presented in the study are included in the article/Supplementary Material, further inquiries can be directed to the corresponding author/s.

## Ethics Statement

This study was approved by Institutional Review Board (IRB) of Jimma University, Institute of Health (IHRPGD/193/18). In addition, the research protocol was approved by the research committee at each hospital before initiating patient enrollment and data collection. All the study protocols were conducted in line with the ethical principle of the Decleration of Helsinki (41). Informed consent (Written or verbal) was obtained from all study participants before commencing patient interview and data abstraction.

## Author Contributions

KF contributed to the conceptualization, study design, data curation, data analysis and interpretation, and manuscript writing. FD, EG, and EA helped with methodology, data curation, drafting, interpretation, supervision, and editing of the data. TM, GF, and LC helped with the methodology, supervision, and editing of the manuscript. All authors critically revised the manuscript and have approved the final version of the manuscript.

## Funding

This work was supported by Jimma University Institute of Health (Grant No. JUIH/193/10). The funding body did not have any role in the study design, data collection and data analysis, interpretation of data, or in writing the manuscript.

## Conflict of Interest

The authors declare that the research was conducted in the absence of any commercial or financial relationships that could be construed as a potential conflict of interest.

## Publisher's Note

All claims expressed in this article are solely those of the authors and do not necessarily represent those of their affiliated organizations, or those of the publisher, the editors and the reviewers. Any product that may be evaluated in this article, or claim that may be made by its manufacturer, is not guaranteed or endorsed by the publisher.
