# Supplementary material for: Management and 30-Day Mortality of Acute Coronary Syndrome in a Resource-Limited Setting: Insight From Ethiopia. A Prospective Cohort Study
Source: Front Cardiovasc Med. 2021 Sep 17;8:707700. doi: 10.3389/fcvm.2021.707700 (PMC8484752; doi:10.3389/fcvm.2021.707700)
Supplement: Supplementary file 1 [file Data_Sheet_1.PDF]

Operational definitions

- Hypertension was considered in patients on antihypertensive medications or repeated blood pressure of 140/90mm Hg or higher.
- Diabetes mellitus was indicted if the patients had previous diagnosis or on antidiabetic medication or if one of the following criteria were recorded during hospital stay: fasting plasma glucose (FPG) test ≥7.0 mmol/l (126 mg/dl), or a random plasma glucose (RPG) test ≥11.1mmol/L (200 mg/dL) with classic symptoms of hyperglycemia, or a Glycated hemoglobin (HbA1c) level ≥6.5%.
- Dyslipidemia was defined as total cholesterol ≥200 mg/dL, LDL-C ≥ 100 mg/dL, triglycerides ≥150 mg/dL, or HDL-C ≤ 40 mg/dL in males and ≤ 50 m
- Myocardial infraction was defined as the presence of previous diagnosis MI irrespective of causes.
- Chronic kidney disease was defined as the presence of kidney damage or glomerular filtration rate (GFR) <60 mL/min/1.73 m^2 for 3 months or more, irrespective of cause.
- HIV/AIDS was indicated by previous diagnosis of HIV irrespective of antiretroviral treatment.
